# Formation of *Trans*-Activation Competent HIV-1 Rev:RRE Complexes Requires the Recruitment of Multiple Protein Activation Domains

**DOI:** 10.1371/journal.pone.0038305

**Published:** 2012-06-04

**Authors:** Dirk Hoffmann, Doreen Schwarck, Carina Banning, Matthias Brenner, Lakshmikanth Mariyanna, Marcel Krepstakies, Michael Schindler, David P. Millar, Joachim Hauber

**Affiliations:** 1 Heinrich Pette Institute – Leibniz Institute for Experimental Virology, Hamburg, Germany; 2 Institute for Clinical and Molecular Virology, University of Erlangen-Nuremberg, Erlangen, Germany; 3 Department of Molecular Biology, The Scripps Research Institute, La Jolla, California, United States of America; International Centre for Genetic Engineering and Biotechnology, Italy

## Abstract

The HIV-1 Rev *trans*-activator is a nucleocytoplasmic shuttle protein that is essential for virus replication. Rev directly binds to unspliced and incompletely spliced viral RNA via the *cis*-acting Rev Response Element (RRE) sequence. Subsequently, Rev oligomerizes cooperatively and interacts with the cellular nuclear export receptor CRM1. In addition to mediating nuclear RNA export, Rev also affects the stability, translation and packaging of Rev-bound viral transcripts. Although it is established that Rev function requires the multimeric assembly of Rev molecules on the RRE, relatively little is known about how many Rev monomers are sufficient to form a *trans*-activation competent Rev:RRE complex, or which specific activity of Rev is affected by its oligomerization. We here analyzed by functional studies how homooligomer formation of Rev affects the *trans-*activation capacity of this essential HIV-1 regulatory protein. In a gain-of-function approach, we fused various heterologous dimerization domains to an otherwise oligomerization-defective Rev mutant and were able to demonstrate that oligomerization of Rev is not required *per se* for the nuclear export of this viral *trans*-activator. In contrast, however, the formation of Rev oligomers on the RRE is a precondition to *trans*-activation by directly affecting the nuclear export of Rev-regulated mRNA. Moreover, experimental evidence is provided showing that at least two protein activation domains are required for the formation of *trans*-activation competent Rev:RRE complexes. The presented data further refine the model of Rev *trans*-activation by directly demonstrating that Rev oligomerization on the RRE, thereby recruiting at least two protein activation domains, is required for nuclear export of unspliced and incompletely spliced viral RNA.

## Introduction

The Rev *trans*-activator of human immunodeficiency virus type 1 (HIV-1) is essential for virus replication (reviewed in [1]). Rev is a small RNA-binding phosphoprotein of 116 amino acids (aa) that constantly shuttles between the nuclear and cytoplasmic compartments of infected cells [2]–[8]. The main function of Rev is to mediate the nuclear export of unspliced and incompletely spliced viral transcripts, either encoding the structural proteins and enzymes Gag, Pol and Env or serving as genomic RNA [9]–[12]. In addition to promoting nuclear RNA export, Rev has also been shown to affect the stability and translation of viral transcripts [10], [13]–[16]. Moreover, more recent studies have provided evidence that Rev also counteracts HIV-1 integration and facilitates the packaging of the viral RNA genome (for recent reviews see [17], [18]).

The structure of Rev is characterized by an organization composed of distinct functional regions. Nuclear import and RNA recognition is mediated by a short stretch of amino acids rich in basic residues [19]–[23]. This RNA binding domain (RBD) spans aa residues 33–46 in Rev’s amino terminal region. A protein activation domain, harbouring a leucine-rich nuclear export signal (NES) at aa residues 78–84, is located in the protein’s carboxy terminal region [24]–[28]. By specifically accessing the cellular CRM1 export pathway, this NES mediates the nucleocytoplasmic translocation of Rev-containing ribonucleoprotein (RNP) complexes across the nuclear envelope [12], [29]–[32]. Finally, sequences flanking the RBD have been demonstrated to constitute a domain required for the formation of homooligomeric Rev complexes [33]–[35]. Detailed biophysical studies suggested that this region forms an amino-terminal amphipathic helix-turn-helix motif [36], [37].

The *cis*-acting target site of Rev on viral RNA is a complex stem-loop structure of 351 nucleotides (nt), termed the Rev Response Element (RRE), that is located in the *env* gene [2]–[5], [38]–[44]. The RRE contains a single, primary, high-affinity Rev binding site, termed stem-loop IIB (SLIIB) [45]–[48]. The initial binding of a single Rev molecule to the SLIIB element initiates the oligomerization of Rev proteins through cooperative assembly along the RRE [41], [49]–[54]. In fact, *in vitro* the occupation of the RRE by as many as 13 Rev molecules has been reported [41], [49]–[51], [53]. The mechanism of the cooperative assembly of Rev monomers on the RRE has been suggested to involve two hydrophobic regions (referred to as “head” and “tail”) in Rev that form a series of symmetrical head-to-head and tail-to-tail protein:protein interactions [35]. Although it is well established that this self-association of Rev on the RRE is required for Rev *trans-*activation [22], [33], [34], [55], [56], it is still unknown how many Rev molecules are sufficient to create a *trans*-activation competent Rev:RRE RNA complex. Attempts to answer this question are hampered by the fact that Rev is characterized by a strong and uncontrolled tendency to self-associate in solution, a process that is not required for Rev function but may compromise the formation of higher Rev:RRE complexes in *in vitro* experiments [47], [57]–[60]. Therefore, we used here cell-based functional studies to analyze in detail the effect of Rev homooligomer formation on *trans-*activation mediated by this essential HIV-1 regulatory protein.

**Figure 1:**
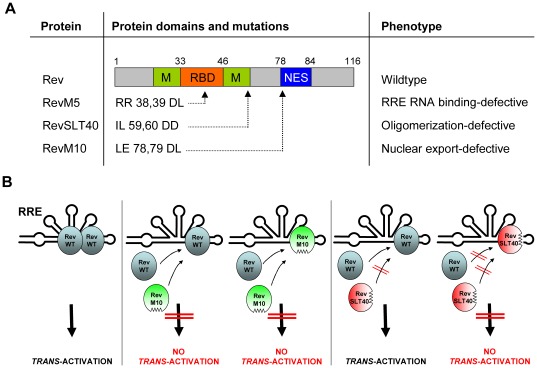
HIV-1 Rev mutants and phenotypes. (A) The RNA binding domain/nuclear localization signal (RBD) is flanked by amino acid sequences that constitute the multimerization interface (M). The nuclear export signal (NES) is located in Rev’s carboxy terminus. The protein name, the positions mutated within the 116-amino acid (aa) Rev protein, the aa changes introduced and the resulting phenotypes are indicated. (B) Schematic representation of Rev:RRE complex formation. Oligomerization of Rev wildtype (WT) molecules on the RRE is essential for *trans*-activation. A nuclear export-defective Rev mutant (RevM10) exerts a more pronounced *trans*-dominant phenotype when compared to an oligomerization-defective protein (RevSLT40). For details see text.

**Figure 2:**
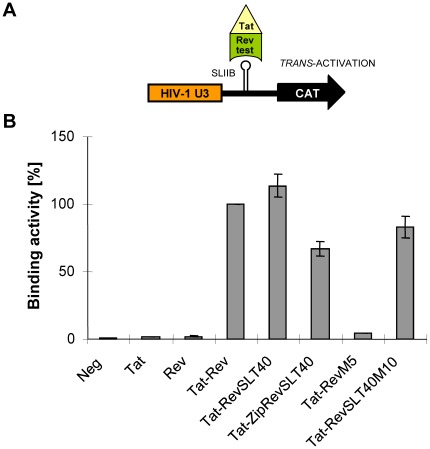
*In vivo* RNA binding by Rev mutants. (A) Schematic representation of the pSLIIB/CAT reporter used to analyze Rev:RRE RNA binding *in vivo*. (B) *In vivo* RRE RNA-binding phenotypes of various *trans*-activators are shown. HeLa cells were transiently cotransfected with the Rev reporter construct pSLIIB/CAT, pBC12/CMV/β-Gal (internal control) and the indicated Tat-Rev fusion constructs. At 48 h post-transfection, CAT and β-Gal expression was analysed by ELISA. CAT values were adjusted for transfection efficiency to the β-Gal level in each culture. Data are expressed as a percentage of wildtype Rev activity (set to 100%).

## Results

### Functional Rescue of a Multimerization-deficient Rev Mutant by Fusion to Heterologous Dimerization Domains

Prior to selecting an oligomerization-deficient Rev mutant for further analyses various functional aspects of Rev had to be considered. The dependence of Rev’s function on the formation of Rev oligomers on the RRE implies that the expression of an oligomerization-deficient Rev mutant should exert a dominant-negative (i.e. *trans-*dominant) phenotype over the wildtype (WT) protein. A classical NES-deficient *trans*-dominant Rev mutant such as, for example, the RevM10 protein [19] (Figure 1A), is able to occupy Rev’s primary binding site SLIIB or, alternatively, can be recruited into nascent Rev homooligomeric complexes on the RRE by protein:protein interaction with wildtype Rev [61] (Figure 1B). In contrast, oligomerization-defective Rev mutants are able to block the SLIIB, but cannot obstruct higher order complexes composed of wildtype Rev molecules (Figure 1B). Thus, when directly compared, the *trans*-dominant phenotype of an oligomerization-defective Rev mutant is less pronounced as opposed to the phenotype of an export-deficient mutant (e.g. RevM10). A frequently overlooked aspect is the fact that Rev mutants that are deficient in their capacity to oligomerize on the RRE have been typically identified *in vitro* using purified components. However, in living cells homooligomer formation by these Rev mutants may still occur. For example, the frequently analyzed RevM4 mutant protein (YSN to DDL at aa position 23,25,26) has been reported to be multimerization-deficient *in vitro* [33], but forms to a significant extent homooligomeric complexes on RRE RNA *in vivo* [55]. In agreement with the latter finding, RevM4 is not able to block Rev function in a *trans*-dominant manner [34], [55], which, as outlined above, should be the case for a *bona fide* oligomerization-defective mutant. Therefore, for our following detailed analyses we selected the previously described mutant RevSLT40, that is characterized by two missense mutations in Rev’s amino-terminal hydrophobic region (IL to DD at aa position 59,60; see Figure 1A) [34]. Importantly, the aa residues I59 and L60 were previously identified by a combination of genetic and biochemical screens as being important for the molecular interactions that mediate the multimeric assembly of Rev on the RRE [35]. Furthermore, in an independent study it has been shown that RevSLT40 is a *trans*-dominant inhibitor of the Rev wildtype protein that is characterized by its inherent inability to form oligomeric complexes on the RRE *in vitro* and *in vivo* [34]. Thus, RevSLT40 fulfills all criteria of an oligomerization-defective Rev protein.

**Figure 3:**
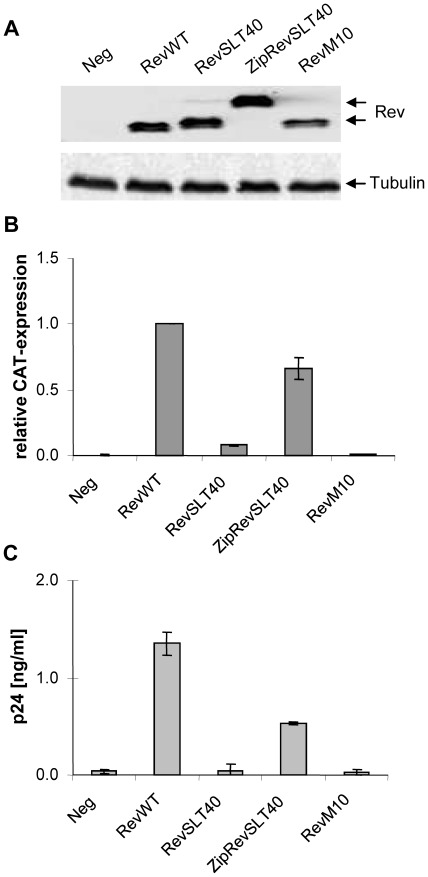
Analysis of Rev *trans*-activation. (A) Expression of Rev wildtype and mutants. HeLa cells were transiently transfected with constructs expressing the indicated Rev constructs and cell extracts were prepared at 48 h post-transfection for Rev- and tubulin-specific (gel loading control) Western blot analysis. (B) Biological activity of various Rev proteins. HeLa cells were transiently transfected with the CAT expressing Rev reporter pDM128/CMV, the indicated Rev expression vectors and pBC12/CMV/β-Gal DNA (internal transfection control). At 48 h post-transfection Rev *trans*-activation was analyzed by CAT-specific ELISA. All CAT values were adjusted for transfection efficiency to the internal β-Gal level and compared to Rev wildtype activity (arbitrarily set to 1.0); WT, wildtype Rev. (C) Biological activity of the indicated Rev proteins as analyzed by provirus rescue assay. HeLa cells were transiently cotransfected with the Rev-deficient proviral DNA pHXB2Δ*rev*, the indicated Rev vectors and the internal control plasmid pBC12/CMV/SEAP. The culture supernatants were collected at 48 h post-transfection and virus particle release was determined by p24^Gag^ antigen ELISA. All p24^Gag^ levels were adjusted to the SEAP activity detected in each culture (transfection control).

**Figure 4:**
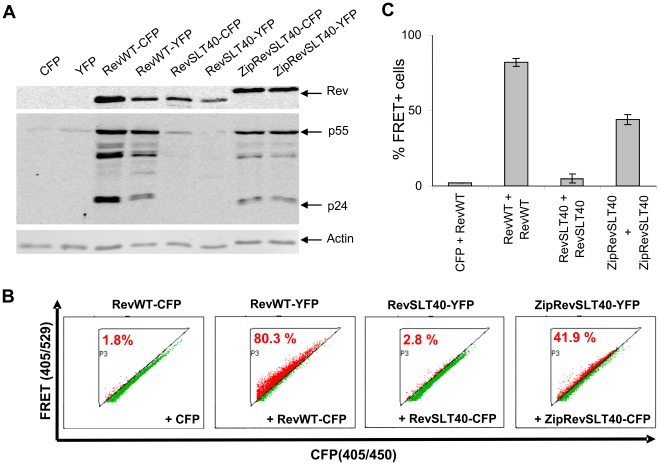
FRET measurement of Rev oligomer formation. (A) Expression level and *trans*-activation capacity of Rev-CFP and Rev-YFP fusion proteins. COS cells were transiently cotransfected with the Rev reporter plasmid pGPV-RRE and the indicated *trans*-activator constructs. At 24 h post-transfection Rev-dependent expression of HIV-1 structural proteins p55^Gag^ and p24^Gag^, the respective Rev *trans*-activators and actin (gel loading control) was detected by Western blot analysis using specific antibodies. (B) Representative FACS plots illustrating the percentage of FRET positive cells. COS cells were transiently transfected with expression vectors for control CFP and RevWT-YFP, or the indicated combinations of Rev donor and acceptor constructs. To provide RRE RNA the plasmid pGPV-RRE was included in each transfection. At 24 h post-transfection cells were harvested and analyzed by flow cytometry for Rev:Rev interaction, represented as FRET positive cells. (C) Mean percentage of FRET-positive cells (adjusted to the background) determined in FRET-FACS experiments.

Rev’s capacity to form oligomers on the RRE may directly affect a distinct Rev activity (e.g. the binding, stabilization, nuclear export or translation of target RNAs). To assess this functional aspect in more detail we initially set out to restore the biological activity of RevSLT40 by engineering a variant in which a dimerization interface was provided by a heterologous sequence. For this, sequences encoding the leucine zipper domain of the yeast transcription factor GCN4, which has been shown to form stable dimers [62], [63], and RevSLT40 were fused.

**Figure 5:**
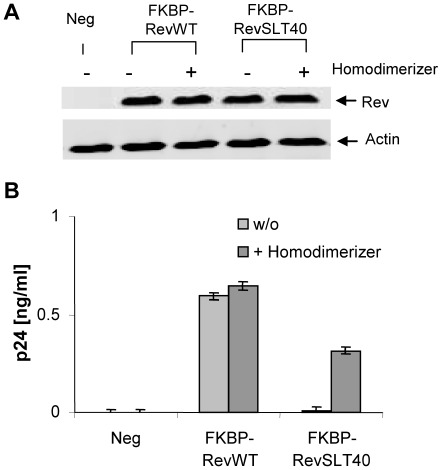
Regulated homodimerization of HIV-1 Rev. (A) Expression of FKBP-Rev fusion constructs. HeLa cells were transiently transfected with plasmids expressing the indicated Rev fusion proteins. Levels of protein expression were determined by Western blot analysis at 48 h post-transfection using specific anti-Rev and anti-actin (gel loading control) antibodies. (B) *Trans*-activation capacity of Rev fusion proteins. A provirus rescue assay was performed by transient cotransfection of HeLa cells with pHXB2Δ*rev* DNA, the internal control plasmid pBC12/CMV/SEAP and the indicated Rev expression vectors in the absence or presence of the chemical homodimerizer AP20187. Culture supernatants were collected at 48 h post-transfection and the accumulation of HIV-1 particles was quantified by p24^Gag^ antigen ELISA. Values of p24^Gag^ antigen were adjusted for transfection efficiency to SEAP activity in each culture supernatant.

We first employed an established reporter gene-based *in vivo* RNA binding assay [55], [64] to confirm that the respective ZipRevSLT40 protein is able to recognize RRE RNA. The pSLIIB/CAT reporter construct contains the CAT gene under the transcriptional control of the HIV-1 LTR promoter (depicted in Figure 2A). The wildtype TAR element, which is the promoter-proximal RNA target sequence of the HIV-1 Tat transcriptional *trans*-activator, is replaced by a sequence encoding the RRE-derived SLIIB high-affinity Rev binding site. This promoter is only activated by Tat-Rev fusion proteins and is not responsive to Tat or Rev alone [64]. Thus, the quantity of CAT produced gives an indication of the RNA binding ability of a given Rev mutant.

**Figure 6:**
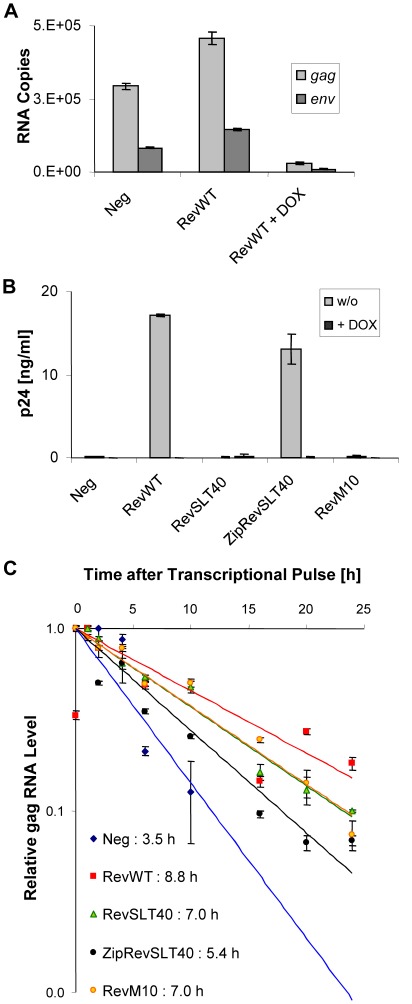
Half-life determination of Rev-regulated transcripts. (A) Expression of Rev-dependent *gag* (unspliced) and *env* (single spliced) mRNAs in HeLa-tTA cells. HeLa-tTA cells were transiently cotransfected with the Rev-deficient doxycyline-regulated proviral construct pUHC-HXB2Δ*rev* and Rev expression plasmid or the parental control vector pBC12/CMV. Transcription of viral RNA was blocked where indicated by 100 ng/ml doxycycline treatment at 5 h post-transfection. Subsequently, the viral RNA levels were quantified at 24 h posttransfection by real-time PCR. Results for viral RNA copies were adjusted to the level of endogenous *gapdh* mRNA. (B) Inhibition of replication by doxycycline treatment. HeLa-tTA cells were transiently cotransfected with pUHC-HXB2Δ*rev,* pBC12/CMV/SEAP (internal control) and the indicated Rev constructs. The accumulation of p24^Gag^ antigen in the culture supernatants was quantified by ELISA at 48 h post-transfection. All values were adjusted for transfection efficiency to the SEAP activity present in each supernatant. Viral expression was blocked when the culture medium was supplemented with 100 ng/ml doxycycline. (C) Effect of the indicated Rev mutants on the half-life of unspliced full-length (*gag*) HIV-1 RNA. Transfection experiments were performed in HeLa-tTA cells using pUHC-HXB2Δ*rev* DNA and the indicated *trans*-activator constructs. Total RNA was isolated and viral *gag* RNA copy numbers were quantified after transcriptional pulse at the indicated time points by real-time PCR and adjusted to the endogenous *gapdh* mRNA level in each sample. The relative RNA levels are show in comparison to the respective initial level (after transcriptional pulse) at time point 0 h (set to 1.0).

**Figure 7:**
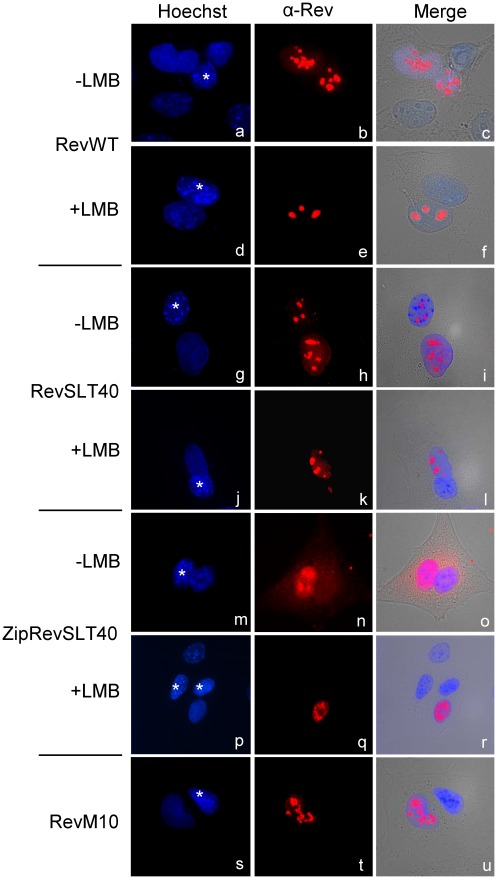
Nucleocytoplasmic shuttling of Rev. Standard interspecies heterokaryon fusion assay for detection of protein nuclear export. HeLa cells were transiently transfected with the indicated Rev expression plasmids and fused at 24 h post-transfection with untransfected NIH3T3 cells (indicated by an asterisk). CRM1-mediated protein shuttling was blocked by treatment of the cultures with leptomycin B (LMB). Localization and nuclear export of Rev proteins was visualized by indirect immunofluorescence microscopy using specific anti-Rev antibody (red label; panel b, e, h, k, n, q, t). Nuclei were stained with Hoechst 33258 (blue label; panel a, d, g, j, m, p, s) and merged with the fluorescence and bright light pictures (panel c, f, i, l, o, r, u).

As expected, the cotransfection of HeLa cells with an unrelated vector or with expression vectors encoding HIV-1 Tat or Rev did not result in *trans-*activation of the pSLIIB/CAT reporter construct (Figure 2B). A detectable level of CAT activity was observed when an expression plasmid encoding a Tat-Rev fusion protein was included in the transfection, serving as positive control. This promoter activation was not detected when the SLIIB binding-incompetent Tat-RevM5 mutant protein [55] was used as negative control. However, the coexpression of constructs carrying the SLT40 mutation (see Figure 1A) clearly resulted in elevated CAT levels, indicating binding of the respective Tat-Rev fusion proteins to the RRE SLIIB site *in vivo*.

**Figure 8:**
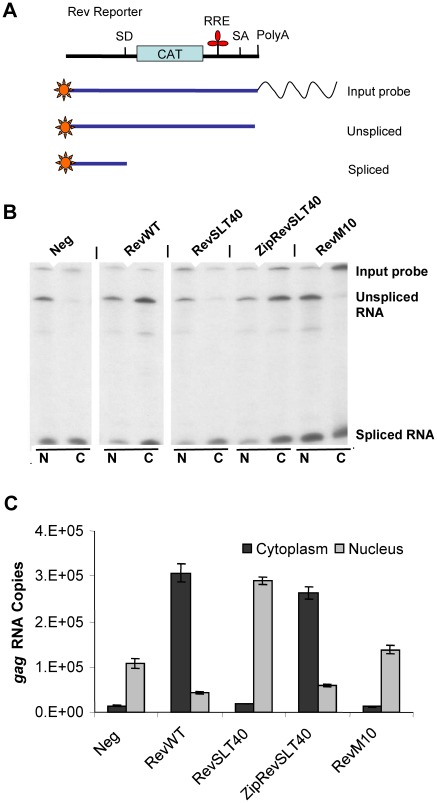
Analysis of Rev-mediated nuclear RNA export. (A) Schematic representation of the nuclease S1 protection assay. The Rev reporter, the end-labelled input probe and the protected sequences, recognizing unspliced and spliced RNA are depicted. (B) Nucleocytoplasmic distribution of Rev-regulated unspliced and spliced RNA determined by nuclease S1 assay. COS cells were transiently cotransfected with Rev reporter DNA pDM128/CMV and the indicated Rev expression vectors. Nuclear and cytoplasmic RNA was isolated at 48 h post-transfection. Unspliced and spliced reporter-derived RNA was detected by autoradiography using the ^32^P-labeled input probe. (N, nuclear RNA; C, cytoplasmic RNA). (C) Quantification of Rev-regulated cytoplasmic and nuclear viral RNA. HeLa cells were transiently cotransfected with pHXB2Δ*rev* and the indicated Rev constructs. At 48 h post-transfection nuclear and cytoplasmic RNAs were isolated and the accumulation of Rev-regulated unspliced *gag* RNA was quantified by real-time PCR. Total viral copy numbers were adjusted to the endogenous *gapdh* mRNA level in each sample.

Further, we analyzed the *trans-*activation capacity of the ZipRevSLT40 fusion protein in various functional assays. First, protein expression in transfected HeLa cells was confirmed by Rev-specific Western blot analysis (Figure 3A). Next, we employed a widely used standard Rev assay that is based on the Rev-responsive reporter construct pDM128/CMV [21], [26]. This construct contains the CAT gene and the RRE sequence, both of which are positioned between HIV-1 splice sites and are under the control of the cytomegalovirus immediate-early promoter. Thus, unspliced CAT encoding transcripts are exported out of the nucleus and subsequently translated in a Rev-dependent manner, resulting in elevated levels of CAT expression. As shown, the nuclear export-deficient control RevM10 as well as the oligomerization-defective mutant RevSLT40, was, as reported previously [19], [34], inactive in this assay (Figure 3B). However, when compared to the biological activity of the Rev wildtype (RevWT) protein the in-frame fusion of the GCN4 leucine zipper to RevSLT40 notably restored Rev function.

**Figure 9:**
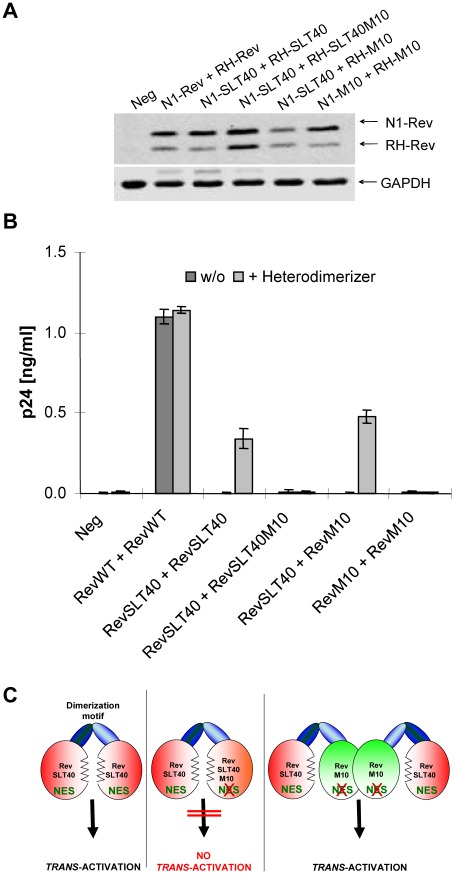
Regulated heterodimerization of HIV-1 Rev. (A) Transient expression of various Rev proteins containing the FKBP (N1) or modified FRAP (RH) heterodimerization motif. COS cells were transiently transfected with the indicated Rev vector constructs, cultured in medium supplemented with the heterodimerizer AP21967, and analyzed by Western blot using specific anti-Rev and anti-GAPDH (gel loading control) antibodies. (B) *Trans*-activation capacity of heterodimeric Rev proteins. COS cells were transiently transfected with the Rev-dependent reporter vector pGPV-RRE, the indicated Rev fusion constructs and pBC12/CMV/SEAP for internal transfection control. Release of HIV-1 particles into the culture supernatant was determined in the absence or presence of the heterodimerizer AP21967 by p24^Gag^ antigen ELISA. For control of transfection efficiency, all p24^Gag^ antigen values were adjusted to the SEAP level present in the supernatant of each culture. (C) *Trans*-activation model of heterodimeric Rev proteins. A Rev dimer needs to exhibit two functional activation domains containing two NES for the biological activity. The remaining ability of RevM10 to multimerize leads to higher order complexes of RevM10:RevSLT40 which contain more than one functional NES and therefore exhibit biological activity.

Since Rev acts in reality on full-length (i.e. unspliced) and incompletely spliced HIV-1 RNA, we next wanted to test Rev *trans*-activation in the viral context. Therefore we cotransfected HeLa cells with the same Rev expression vectors as before, together with a HIV-1 proviral DNA that was mutated in the *rev* gene (HIVΔ*rev*) [65]. The capacity of the various Rev mutants to rescue in *trans* the replication of this defective provirus was subsequently analyzed by monitoring the accumulation of HIV-1 p24^Gag^ antigen in the respective culture supernatants. The data that were obtained in these experiments closely resembled the results that were obtained by using the more artificial CAT reporter construct (Figure 3B). Again, the RevSLT40 or RevM10 mutants were inactive in this assay, while expression of the ZipRevSLT40 fusion protein resulted in the release of detectable amounts of virus particles, although not to the same extent as seen with RevWT (Figure 3C).

To directly confirm leucine zipper-dependent interaction of Rev in transfected cells we employed a flow cytometry-based FRET assay system [66]. COS cells were transiently transfected with vectors encoding Rev fusion proteins that were tagged at their carboxy terminus either with a donor or an acceptor fluorophore (CFP or YFP, respectively). The transfection protocol also included the GPV-RRE reporter vector, that contains the HIV-1 *gag-pol* gene and expresses Gag proteins in a Rev-dependent fashion [67]. At 24 h post-transfection, Western blot analyses revealed the expected Rev phenotypes. Significant amounts of Gag proteins were only detectable in the cultures in which either RevWT or ZipRevSLT40 proteins were expressed, but not in case of RevSLT40 (Figure 4A). FACS-FRET analysis on cell cultures which were transfected in parallel revealed that coexpression of CFP and YFP labelled RevWT resulted in 80.3% of FRET positive cells which fell within the background adjusted gate, indicating pronounced Rev:Rev interaction (Figure 4B and 4C). As expected, the RevSLT40 mutant did not produce a significant FRET signal (2.8%), while expression of ZipRevSLT40 yielded 41.9% of FRET positive cells.

Finally, to further support the preceding results we also wanted to confirm the reconstitution of Rev activity by using another independent technical approach. The ARGENT regulated homodimerization kit (ARIAD Pharmaceuticals) employs cell permeant ligands (e.g. the chemical homodimerizer molecule AP20187) that allow the induction of dimerization of proteins that contain the FK binding protein (FKBP) motif (e.g. FKBP-Rev fusions). Therefore we generated FKBP-RevWT and FKPB-RevSLT40 encoding expression vectors and analyzed them as before (Figure 5A and 5B). These experiments confirmed that the formation of RevSLT40 homodimers is able to rescue replication of HIV-1 to a level of ∼50% of RevWT activity. Obviously, this effect was only observed in presence of the chemical homodimerizer (Figure 5B).

Taken together, the combined data suggest that the biological activity of an otherwise oligomerization-defective Rev mutant can be restored to a significant extent by in-frame fusion of this mutant protein to a heterologous dimerization motif.

### Dimerization Permits Nuclear Export of Rev-regulated Transcripts

Next we set out to identify distinct Rev activities that depend on the oligomerization of Rev. Since Rev has been reported to affect the stability of viral mRNA, we first examined the effect of Rev and sealected Rev mutants on the half-life of RRE-containing messages by employing the transcriptional pulsing approach using the tetracycline-regulated promoter system [68]. This experimental system allows assessment of the stability of specific mRNA in the absence of general transcription inhibitors such as actinomycin D, a drug that can interfere with nucleocytoplasmic shuttling [69].

HeLa-tTA cells constitutively expressing the tetracycline-sensitive *trans*-activator (tTA) were transiently transfected with a derivative of the HIVΔ*rev* proviral DNA (pUHC- HIVΔ*rev*), in which the viral 5′-LTR was replaced by the tTA-responsive promoter, thereby allowing doxycycline-induced transcriptional shutoff. As detected by real-time PCR, *env* and more pronounced *gag* transcripts were more abundant in RevWT coexpressing cells (Figure 6A). Only residual amounts of *gag* and *env* mRNA copies were detected when the culture supernatant was supplemented with 100 ng/ml of doxycycline. A parallel experiment in which p24^Gag^ antigen levels were monitored in the supernatants of the transfected cultures demonstrated the expected phenotypes, particularly RevWT- and ZipRevSLT40-dependent HIV-1 particle release in absence of doxycycline treatment (Figure 6B). Finally, upon transcriptional shutoff the quantification of *gag* sequences revealed, as previously reported [10], [11], Rev-dependent stabilization of these *gag* transcripts. Overall, *gag* mRNAs were differentially stabilized by various Rev proteins. However, the ability of Rev to form oligomers clearly did not correlate with Rev-mediated mRNA stabilization (Figure 6C). Thus, failure of Rev to form protein:protein oligomers does not significantly affect the stability of Rev-regulated transcripts and can therefore not explain the observed inactivity of RevSLT40 in the more general *trans*-activation assays (Figure 3).

To directly analyze the nuclear export ability of RevSLT40 and ZipRevSLT40, standard heterokaryon assays were performed, in which transiently transfected human HeLa cells (expressing the various Rev proteins) were fused with untransfected mouse NIH 3T3 cells to form heterokaryons. Fixed cells were analyzed by indirect immunofluorescence microscopy using Rev-specific antibodies. The respective cultures were also treated with Hoechst 33258 dye, which allows discrimination of human and mouse nuclei (the latter are characterized by a typical speckle pattern). Clearly, RevWT protein was able to migrate from the human to the mouse nucleus in these experiments (Figure 7, panel a–c). As expected, exposure of the cell culture to leptomycin B (LMB), a highly specific small-molecular weight inhibitor of the CRM1 nuclear export receptor [70], [71], abolished Rev’s ability to exit the human nucleus (Figure 7, panel d–f). A comparable result was also observed in a control experiment when the export-deficient mutant RevM10 was analyzed (Figure 7, panel s–u). Interestingly, both the oligomerization- and *trans*-activation-deficient mutant RevSLT40, as well as the ZipRevSLT40 protein, displayed comparable CRM1-dependent nuclear export activities in this type of assay (Figure 7, panel g–l and panel m–r, respectively). These data suggested that, in absence of RRE RNA, oligomerization of Rev is not required for nuclear export of Rev proteins via the CRM1-pathway.

Rev-dependent nucleocytoplasmic distribution of RRE-containing mRNA was next monitored by using two different experimental designs. First, COS cells were transiently cotransfected with the Rev-responsive reporter construct pDM128/CMV and various Rev expression plasmids. At 48 h post-transfection nuclear and cytoplasmic RNA was collected from the transfected cell cultures and analyzed by quantitative S1 nuclease protection analyses. The endlabelled input probe allowed its discrimination from unspliced and spliced reporter mRNA (depicted in Figure 8A). Inspection of the obtained data revealed that the oligomerization-defective mutant RevSLT40 failed to mediate efficient cytoplasmic accumulation of unspliced mRNA (Figure 8B). Obviously, nuclear export of these RRE-containing transcripts was restored when the ZipRevSLT40 fusion protein was coexpressed, indicating that oligomer-formation of Rev is required for the translocation of Rev-regulated transcripts across the nuclear envelope.

These results were confirmed in an independent and more quantitative assay. HeLa cells were again contransfected with the full-length proviral DNA HIVΔ*rev* together with selected Rev expression vectors. Unspliced *gag*-specific messages were subsequently detected by quantitative real-time PCR in cytoplasmic and nuclear RNA, isolated from the transfected cultures at 48 h post-transfection. The fractionation method was controlled by detection of the nuclear U6 snRNA using a slot-blot device (not shown). This analysis also demonstrated pronounced cytoplasmic accumulation of HIV-1 *gag* mRNA in a Rev-dependent fashion (Figure 8C). In agreement with the data raised above, RevSLT40 was incapable of mediating the nuclear export of *gag*-specific transcripts while, in sharp contrast, fusion of the GCN4 leucine zipper to RevSLT40 clearly restored this Rev-specific activity. Furthermore, control experiments in which the cotransfection of a Rev expression vector was omitted, or in which nuclear export-deficient RevM10 protein was expressed, resulted in the expected negative phenotype (Figure 8C).

In sum, these data demonstrate that oligomerization of Rev is essential for Rev-mediated nuclear export of RRE-containing viral transcripts.

### Rev *Trans*-activation Requires Multiple Activation Domains

To address the question of whether Rev has to oligomerize in order to recruit multiple NES-containing protein activation domains onto the RRE RNA, another version of the ARGENT dimerization kit (ARIAD Pharmaceuticals) was used. This system employs a specific cell permeant heterodimerizer molecule, the rapamycin analog AP21967, which allows the dimerization of two different proteins when they are attached to appropriate binding domains. Therefore, various Rev encoding sequences were fused to engineered versions of either the FKBP motif (e.g. N1-Rev) or a domain of the PI3K homolog FRAP (e.g. RH-Rev). Rev protein expression by these constructs was subsequently confirmed by transient transfection of COS cells and Western blot analysis (Figure 9A). The transfection of matched pairs of these expression vectors also allowed the formation of Rev heterodimers when the culture medium was supplemented with AP21967. In addition, inclusion of the Gag-expressing GPV-RRE reporter vector into the transfection protocol permitted monitoring of Rev activity. As shown, the fully oligomerization competent RevWT protein displayed the highest activity in this assay, while homooligomers formed by the nuclear export-deficient mutant RevM10 were, as expected, completely inactive (Figure 9B). Interestingly, RevSLT40 homodimers, as well as heterodimers formed by the oligomerization-defective mutant RevSLT40 and RevM10 were to a certain extent also active. The latter result can be explained by RevM10’s remaining intrinsic capacity to form homomultimers [33], [55]. Thus, in presence of the heterodimerizer molecule, higher order RH-RevM10 complexes may recruit additional N1-RevSLT40 proteins (and thereby functional activation domains; depicted in Figure 9C). Therefore, the double mutant RevSLT40M10 was also included in these analyses. Clearly, heterodimers composed of N1-RevSLT40 and RH-RevSLT40M10 were completely devoid of any biological activity in these experiments (Figure 9B), indicating that at least two activation domains are required for Rev-mediated *trans*-activation.

## Discussion

The translocation of unspliced and incompletely spliced viral RNAs, from their nuclear site of transcription to the cytoplasm, the site of translation and virus assembly, is mediated by the Rev *trans*-activator protein [9]–[12]. In the nucleus, Rev directly recognizes viral RNA via the *cis*-acting RRE sequence, multimerizes, and interacts with CRM1 for subsequent nuclear exit of the Rev-bound transcripts (reviewed in [1], [72], [73]). Accordingly, in recent years a large body of studies focused on Rev’s RNA binding properties and, particularly, on its role in nuclear export. In comparison, relatively little attention was focused on Rev’s capacity to form homooligomeric complexes on the RRE and the role of this process in Rev *trans*-activation.

In this study, by employing various Rev *trans*-activation assays, including assays measuring Rev-dependent HIV-1 particle release, we were able to provide evidence that multimerization of Rev on the RRE is required for nuclear export of Rev-regulated viral mRNA. Importantly, this was demonstrated by an experimental gain-of-function approach that allowed the recording of positive and therefore meaningful results. The fusion of heterologous dimerization motifs to the oligomerization-deficient mutant RevSLT40 restored not only *trans-*activation in the respective assays (Figure 3), but also reconstituted Rev-mediated nuclear mRNA export, which was reflected by Rev dimerization-dependent cytoplasmic detection of RRE-containing transcripts (Figure 8). These data are in agreement with a previous study that reported that oligomerization of Rev is important for nuclear export of Rev:RRE complexes [56]. However, the evidence supporting this notion was based on negative data (i.e. loss-of-function) using a single reporter construct whose readout is sensitive to many of Rev’s established activities (e.g. RNA stabilization, nuclear export, translation) and no direct RNA transport data were provided. Moreover, other previous studies, proposing that multimerization-deficient Rev mutants are defective in the nuclear export of unspliced RRE-containing mRNAs [19], [33] employed the RevM4 mutant, that is, as outlined before, characterized by an inconsistent oligomerization phenotype [33], [55]. Therefore, by using the multimerization deficient mutant RevSLT40 [34], the data obtained in the present study provide to our knowledge the so far most definitive experimental evidence that multimer-formation of Rev is essential for nuclear export of Rev-containing RNP complexes.

Clearly, dimer induction by fusion of heterologous dimerization motifs to the amino terminus of the otherwise multimerization-deficient mutant RevSLT40 could not have been expected to restore Rev activity to its full extent. Nevertheless, *trans*-activation activity was routinely restored to levels ranging from 40% to 68% of Rev wildtype activity, depending on the assay used (see Figure 3 and 5). This is in good agreement with our quantitative FRET measurements (Figure 4), in which co-expression of CFP and YFP labeled ZipRevSLT40 resulted in a dimerization capacity of about 52% when compared to the wildtype protein (RevWT: 80.3% FRET positive cells; ZipRevSLT40∶41.9% FRET positive cells).

Although in our experiments the formation of a Rev dimer was sufficient to drive to a detectable extent virus replication, it is still unknown how many Rev molecules have to be assembled on the RRE for *maximal* activity. A large body of previous biochemical and cell-based studies resulted in a model of Rev:RRE interaction, in which the initial binding of a Rev monomer to the SLIIB high-affinity binding site triggers the recruitment of additional Rev monomers by cooperative Rev:Rev interaction, resulting in the occupation of secondary low-affinity sites in the RRE [41], [49], [50], [74]. Careful protein titration studies in combination with functional assays indicated already previously that two monomeric Rev proteins bound to the RRE results in measurable biological activity [75]. Similar data were also reported in an independent functional study using a heterologous Rev-responsive reporter construct. Considerable Rev activity was observed when the RRE was replaced by phage MS2 operator RNA and Rev was expressed as fusion to MS2 coat protein [76]. Apparently, upon RNA binding the Rev/MS2 protein dimerized via its MS2 moiety [77], resulting in Rev *trans-*activation. Rev activity then further increases with the number of Rev molecules that are recruited onto the RRE [33], [41]. Importantly, this sequential model of Rev:RRE interaction has been recently confirmed and refined by employing single-molecule fluorescence spectroscopy, directly demonstrating that Rev indeed assembles on RRE RNA one molecule after another [52].

HIV-1 Rev *trans*-activation induces the transit from the early phase to the late phase of viral mRNA expression, a process that depends on Rev’s nuclear mRNA export activity [1], [72], [73]. The transport of Rev-containing RNP complexes across the nuclear envelope is mediated by the cellular export receptor CRM1 and associated factors [29]–[32]. CRM1 generally mediates the nuclear export of non-coding RNA, ribosomal subunits and numerous proteins and therefore specifies a rather uncommon route for the nuclear export of mRNA [78], [79]. By using the highly specific CRM1 inhibitor leptomycin B [70], [71], the analysis of Rev shuttling clearly demonstrated nuclear export of the oligomerization-deficient RevSLT40 protein via the CRM1 pathway (Figure 7). In contrast, however, RevSLT40 was unable to mediate the export of RRE-containing transcripts, an activity that was restored by dimer formation (Figure 8). This finding may suggest that a single CRM1 molecule is sufficient to mediate the nuclear export of a monomeric Rev protein in absence of RRE RNA-binding. However, multiple Rev proteins and, due to Rev NES:CRM1 interaction [80], possibly an equivalent number of CRM1 molecules have to be recruited onto RRE-containing transcripts for their efficient nucleocytoplasmic translocation. Clearly, this notion is in agreement with the results obtained by using Rev heterodimers, suggesting that at least two Rev NES are required for the formation of an export-competent Rev:RRE complex (Figure 9).

A somewhat alternative interpretation of the presented data may be that Rev oligomerization on the RRE is not only important for recruitment of multiple CRM1 proteins, but also provides a binding platform for the recruitment of additional host factors that facilitate the nuclear export of RRE-containing transcripts. Among others the most likely candidates for such Rev cofactors are some RNA helicases, particularly the DEAD box protein 1 (DDX1) [81], the human Rev-interacting protein (hRIP) [82], [83], eukaryotic initiation factor 5A (eIF5A) [84], [85], and Src-associated protein in mitosis of 68 kDa (Sam68) [86], [87]. Multiple lines of evidence demonstrated that these host factors directly or indirectly interact with Rev and critically participate in the posttranscriptional processing of Rev-regulated mRNA (reviewed in [88]–[91]). Importantly, by employing total internal reflection fluorescence (TIRF) microscopy, a recent study demonstrated that particularly the DEAD box protein DDX1 promotes Rev oligomerization on the RRE [92]. Thus, the formation of homooligomeric Rev complexes on the RRE is not only facilitated by cellular proteins such as DDX1, but may also be a precondition for the recruitment of one or more of the host factors mentioned above into nascent Rev-containing RNP.

In sum, the data presented here directly demonstrate that Rev oligomerization on the RRE, and thereby the recruitment of multiple NES-containing Rev activation domains, is required for nuclear export of unspliced and incompletely spliced viral RNA. Because Rev-mediated RNA export is essential for virus replication, the interference with Rev activity [90], [93], for example by blocking Rev’s capacity to self-associate on the RRE, may represent an attractive target for the development of novel antiretroviral therapies.

## Materials and Methods

### Molecular Clones

The plasmids pcRev and pcTat contain the cDNA of the retroviral genes derived from HIV-1 strain HXB3 and have been described elsewhere [94]. The parental vector pBC12/CMV was used as a negative control in all transfections [95]. The *trans*-dominant mutants pcRevSLT40 and pcRevM10 have been described previously [19], [34]. The pcZipRevSLT40 construct was generated by inserting the cDNA from the yeast GCN4 leucin zipper motif (NH_2_-MDPKLQRMKQLEDKVEELLSKNYHLENEVARLKKLVGG-COOH) [62], [63] at the 5′ end into the reading frame of the RevSLT40 cDNA. The constructs expressing Tat-Rev fusion proteins pcTat/Rev, pcTat/RevM5, pcTat/RevM10 and pcTat/RevSLT40 have been described in detail elsewhere [34], [55]. The plasmid encoding for Tat-ZipRevSLT40 was constructed by inserting the leucine zipper motif into the pcTat/RevSLT40 expression plasmid. RevSLT40M10 expression constructs were generated by introducing the RevM10 (LE 78,79 DL) mutation into the RevSLT40 cDNA by using PCR site-directed mutagenesis. The plasmids encoding for the regulated dimerization proteins FKBP-Rev and FKBP-RevSLT40 were constructed by inserting the cDNA of the homodimerization motifs from the plasmid pC4-Fv1E (ARIAD Pharmaceuticals) at the 5′-end into the reading frame of respective Rev expression plasmids. The expression plasmids encoding for N1- and RH-Rev fusion proteins were generated by subcloning the heterodimerization motifs from the parental vectors pC_4_EN-F1E and pC_4_-R_H_E (ARIAD Pharmaceuticals) into the respective Rev expression plasmids. The RRE RNA binding reporter plasmid pCAT/SLIIB contains a LTR promoter in which the *trans*-activator response element (TAR) was replaced by the Rev primary binding site, stem loop II, derived from the Rev response element (RRE) [64]. The plasmids pDM128/CMV [21], [26], pGVP-RRE [67] and the proviral construct pHXB2Δ*rev* [96] are previously established Rev reporter constructs. The doxycycline-regulated pUHC-HXB2Δ*rev* expression plasmid was generated by replacing the viral 5′-LTR promoter through the tet-off promoter derived from the pUHD13-3 plasmid [97]. The expression plasmids pBC12/CMV/β-Gal and pBC12/CMV/SEAP were used for internal transfection control [98], [99]. The constructs encoding for Rev-CFP and Rev-YFP fusion proteins were generated by fusing the respective Rev cDNAs in-frame to the 5′ end of the fluorescent protein coding region in the parental vector pECFP-N1 and pEYFP-N1 (Clontech) [66].

### Cell Culture, Transfections, and Assays

HeLa (ATCC Cat.# CCL-2), HeLa-tTA [97] (obtained from Prof. Wolfgang Hillen, University Erlangen-Nürnberg) and COS (ATCC Cat.# CRL-1650) cells were cultured in Dulbecco’s modified eagle medium (DMEM) supplemented with 10% fetal bovine serum and were transiently transfected either with TurboFect (Fermentas) or TransIT (Mirrus) according to the manufacturers’ protocol or with DEAE dextran as previously published [64]. Routinely, 2×10^5^ cells were transfected with 500 ng reporter plamid (pDM128/CMV, pSLIIB/CAT, pGPV-RRE, pHXB2Δ*rev* or pUHCHXB2Δ*rev*, respectively), 250 ng Rev construct and 125 ng plasmid DNA for internal transfection control (pBC12/CMV/SEAP or pBC12/CMV/β-Gal). At 48 h post-transfection, culture supernatants were removed and analyzed for particle release by p24^Gag^ antigen ELISA (Innogenetics NV) and adjusted to the secreted alkaline phosphatase (SEAP) level (internal transfection control). Cell pellets were harvested by trypsin and cell extracts were prepared using E1A lysis buffer (50 mM Hepes pH 7.5, 150 mM NaCl, 1% NP-40). Western blot analyses were performed by using anti-HIV-1 p55^Gag^ specific antibodies (NIH AIDS), specific anti-Rev specific antibodies [20], anti-tubulin (Sigma), anti-GAPDH (Santa Cruz) and anti-actin (Sigma). Reporter expression of pDM128/CMV and pSLIIB/CAT was measured with the CAT ELISA Kit (Roche) in accordance to the manufacturer’s protocol. The CAT expression levels were adjusted to the expression level of the internal transfection control β-Gal. To induce the regulated dimerization, 500 mM of AP20187 (ARIAD Pharmaceuticals) for homodimerization and 500 mM of AP21967 (ARIAD Pharmaceuticals) for heterodimerization was added 5 h post-transfection by exchanging the culture media. For FACS-based FRET analyses, 5×10^5^ COS cells were transiently transfected with 500 ng of the various Rev acceptor and donor constructs together with 500 ng of the pGPV-RRE reporter. Western blot analyses and FACS measurement were performed 36 h posttransfection. For heterokaryon assays, 2×10^5^ HeLa cells were transiently transfected with 1 µg of the respective Rev expression plasmids.

### FACS-FRET

For analyses of Rev-Rev interactions, FRET signals of COS cells, transfected with Rev donor and acceptor plasmids, were measured with the FACSAria system (BD Bioscience) using the 405 nm and 488 nm laser lines as previously described [66]. The CFP-positive COS cells were excited with the 405 nm laser and fluorescence was analyzed in the CFP channel by using the standard 450/40 filter (Semrock). YFP positive cells were excited with the 488 nm laser and measured with the 529/24 filter (Semrock). The FRET-positive cells were monitored by 488 nm excitation and signal emission was detected with the 529/24 filter in the YFP channel. For each sample at least one thousand CFP/YFP positive cells were analyzed, and signals were adjusted to the background.

### RNA Isolation and PCR

Total cellular RNA was isolated with the TriFAST system (PeqLab) according to the manufacturer’s protocol. For the isolation of cytoplasmic and nuclear RNA cells were fractionated into the subcellular compartments by using Nonidet-P40 buffer (10 mM Hepes pH 7.8, 10 mM KCl, 20% glycerin, 0.25% Nonidet-40 and 1 mM DTT) as described before [100]. To remove DNA plasmid contaminations, samples were treated with 1 U DNase (Promega) and subsequently RNA was phenol/chloroform/isoamyl alcohol extracted. For analyses of viral RNA distribution and total RNA expression, 1 µg of RNA was reverse transcribed using the M-MLV reverse transcriptase (Promega) and poly dT primer in accordance with the manufacturer’s protocol. For the quantification of viral RNA, corresponding cDNAs were used for real-time PCR analyses. Copies of viral RNA were determined by using a plasmid standard. The viral RNA copies were adjusted to the cellular *gapdh* RNA level. The following oligonucleotides and Taqman probes were used for RNA detection: *gag/env* forward, 5′–GCAGGACTCGGCTTGCTGAA–3′; *gag* reverse, 5′–AGGATTAACTGCGAATCG TTCTA–3′; *gag* probe, 5′–(FAM)–TTGACTAGCGGAGGCTAGAAGGAGA–(TAMRA)–3′; *env* reverse, 5′–GCTACTACTAATGCTACTATTGCT–3′; *env* probe, 5′–(FAM)–ATAGAGAAGCTTGATGAGTCTGACTGTT–(TAMRA)–3′; *gapdh* forward, 5′-GTCATC AATGGAAATCCCATCA-3′; *gapdh* reverse, 3′-TGGTTCACACCCATGACGAA-5′; *gapdh* probe, 5′-(FAM)-TCTTCCAGGAGCGAGATCCCTC-(TAMRA)-3′. Nuclease S1 protection assay was performed as described elsewhere [98]. The radiolabeled probe includes the HIV-1 splice site derived from the pDM128/CMV plasmid [21] and heterologous non-HIV sequences to distinguish between the input probe and the unspliced as well as the spliced RNA-DNA hybrids.

### RNA Stability Measurements

For analyses of the influence of Rev mutants on the stability of viral RNA, HeLa-tTA cells were transiently transfected with the doxycycline-regulated construct pUHC-HXB2Δ*rev* and the respective Rev expression plasmids. To determine the application of the pUHC-HXB2Δ*rev* construct, HeLa-tTA cells were cultured 5 h post-transfection with 100 ng/ml doxycycline. At 24 h posttransfection total RNA was isolated and the copies of viral unspliced *gag* RNA and single-spliced *env* RNA was quantified by real-time PCR. To determine the half-life of viral *gag* RNA in the presence of Rev mutants, the transcriptional pulsing strategy was employed [68]. Therefore transiently transfected HeLa-tTA cells were incubated with 100 ng/ml doxycycline and reporter RNA transcription was blocked over night. On the next day the transcriptional pulse was induced by changing the media to fresh DMEM lacking doxycycline for additional 4 h of culturing. Viral RNA transcription was stopped by treating the cultures with various concentrations of doxycycline. Total cellular RNA was isolated at time points 0, 1, 2, 4, 6, 10, 16, 20 and 24 h, and *gag* RNA expression was quantified by real-time PCR.

### Heterokaryon Assay

To analyze the nucleocytoplasmic shuttling of Rev, 6×10^4^ HeLa cells, transiently transfected with various Rev expression plasmids, were cocultured at 24 h post-transfection with 8×10^4^ murine NIH3T3 cells (ATCC Cat.# CRL-1658) on coverslips. The next day, protein expression was blocked by using 100 µg/ml cycloheximide and 20 nM leptomycin B (LMB) to inhibit CRM-1 specific nuclear export. Cell fusion was performed by using PEG1500 (Roche) according to the manufacturer’s protocol and cells were cocultured in cycloheximide- and LMB-containing medium for an additional hour. Rev protein shuttling was monitored by indirect immunofluorescence microscopy.

### Immunofluorescence Microscopy

To analyze the nucleocytoplasmic shuttling of Rev mutants, human:mouse hybrid cells were grown on coverslips and fixed with 4% paraformaldehyde. Subsequently, cells were permabilized with 0.1% Triton X-100 and stained with a mouse monoclonal anti-Rev antibody, followed by a Cy3-conjugated anti-mouse antibody [20]. Nuclei were visualized by Hoechst 33258 staining. The cells were mounted in mowiol medium and analysed using a Zeiss Axiovert-200 M microscope.
